# Transcriptome analysis of long noncoding RNAs reveals their potential roles in anthracycline-induced cardiotoxicity

**DOI:** 10.1016/j.ncrna.2022.01.002

**Published:** 2022-01-23

**Authors:** Nhan Nguyen, Terezinha Souza, Jos Kleinjans, Danyel Jennen

**Affiliations:** Department of Toxicogenomics, GROW School for Oncology and Developmental Biology, Maastricht University, Maastricht, the Netherlands

**Keywords:** anthracycline, lncRNA, Cardiotoxicity, RNA sequencing, ImpulseDE2, ANT, Anthracycline, lncRNA, long non-coding RNA, DOX, doxorubicin, EPI, epirubicin, IDA, idarubicin, DE, differentially expressed, Log2FC, log2 fold change

## Abstract

**Aims:**

Anthracyclines (ANTs) are essential chemotherapeutic agents; however, their adverse effects can lead to heart failure in cancer survivors. While long non-coding RNAs (lncRNAs) have become new players in cellular processes, there is limited knowledge on lncRNA expression related to anthracyclines-induced cardiotoxicity. This study investigates the lncRNA profiles in human cardiac microtissues exposed to 3 popular ANTs, namely doxorubicin, epirubicin, and idarubicin, as well as in heart biopsies from ANT-treated patients.

**Methods and results:**

The *in vitro* microtissues were exposed to each ANT at 2 doses over 2 weeks; the transcriptome data was collected at 7 time points. The human biopsies were collected from heart failure patients who underwent ANT treatment and control subjects. Over 100 lncRNAs were differentially expressed in each *in vitro* ANT treatment condition compared to control samples; 16 of them were differentially expressed across all ANT-treated conditions. The lncRNA databases and literature revealed insight on how these lncRNAs relate to heart failure and cellular functions. For instance, *H19* and *RMRP* are involved in heart failure progression, while *BDNF-AS* is a cardiomyocyte damage-associated gene; *SNHG7* is a cardiac hypertrophy regulator. *PCAT19* can promote the miR‐182/PDK4 axis and modulate p53 expression, whereas *SNHG29* can regulate the Wnt/β-catenin signaling pathway via the miR-223–3p/CTNND1 axis. Other lncRNAs, which were only differentially expressed in particular ANT-treated conditions, are also involved in cardiomyocyte damage and heart failure disease. The alterations of these lncRNA expressions in the *in vitro* cardiac tissue were also affirmed by similar changes in the human biopsies.

**Conclusion:**

This study revealed several lncRNAs that can be potential biomarkers or targets for further ANT-induced cardiotoxicity investigation, according to the transcriptome in both human cardiac microtissues expose to ANTs as well as in heart biopies form ANT-treated patients. Especially, *H19* lncRNA showed its contribution to on-target toxicity, in which it is involved in both chemoresistance and cardiotoxic mechanism.

## Graphical abstract

**Figure:**
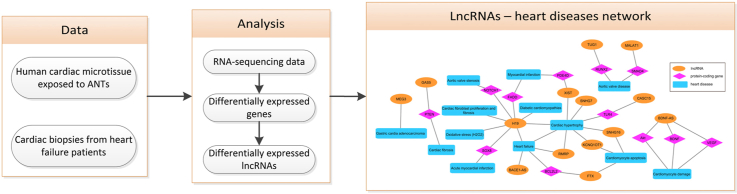

## 1 Introduction

Heart failure is a clinical syndrome, which is not only caused by cardiovascular disorders but also by clinical intervention. Some drugs can expedite the pathology of cardiovascular patients, while other drugs may cause heart failure in patients without heart disease comorbidities [1]. Although these drugs can lead to undesirable effects, therapeutic alternatives have not been offered, especially in oncology. All modalities of cancer therapy can adversely impact the cardiovascular system [2]. For instance, anthracyclines (ANTs) form an essential drug family in chemotherapy, but are also cardiotoxic agents which provoke heart failure in cancer survivors [3]. While three ANTs, namely doxorubicin (DOX), epirubicin (EPI), and idarubicin (IDA), are commonly used in cancer treatment [3], the optimal strategy to prevent their cardiotoxicity remains unknown [4]. Understanding the adverse mechanism of these ANTs may assist the medication as well as limit the drug side effects. Researchers have proposed that ANTs can inhibit topoisomerase 2β and produce excessive reactive oxygen species, which can activate cell death pathways and damage the mitochondrial and the cell structure respectively [3,5]. Recent research has shown that ANTs can alter signaling pathways, which are related to cardiomyocyte survival and cardiac inflammation [6]. However, further investigation is still crucial to clarify the ANT cardiotoxic mechanisms.

Investigating gene expression plays a key role in elucidating the underlying cellular mechanisms of action. Together with protein-coding genes, non-coding RNA genes such as rRNAs, tRNAs, and miRNAs are involved in cellular regulations. Recently, long non-coding RNAs (lncRNAs), which are non-coding RNAs longer than 200 nucleotides, have gained widespread attention as new players in numerous cellular functions such as transcription regulation, mRNA stability, translation regulation, and post-translational modifications [7,8]. Several studies have indicated that lncRNAs can provide a detailed view of cardiac development and pathology. For instance, 1146 lncRNAs in mouse hearts were differentially expressed between postnatal day 1, day 7, and day 28; a particular lncRNA, Gm12245-201 (ENSMUST00000117266) [9], showed its potential in cardiomyocyte proliferative activity and cardiac hyperplastic-to-hypertrophic growth transition [10]. Another study emphasized the important function of other lncRNAs such as *H19*, *MALAT1*, and *MDNCR* at different stages of human cardiac development and heart disease [11], which inform the lncRNAs’ abilities becoming potential biomarkers as well as therapeutic targets in heart diseases [12]. While some studies have demonstrated the lncRNA functions in pathology, other studies have conceded the lncRNA expression patterns related to treatment mechanisms. An investigation in non-alcoholic fatty liver disease revealed the pharmaceutical mechanisms of berberine in both mRNAs and lncRNAs [13]. Another study in Ang II-treated cardiac fibroblasts also showed the significantly altered expression of lncRNAs, such as NR024118 [14]. These researches emphasize that lncRNAs involve not only in disease pathology but also in the cell responses to drug treatment.

As a popular drug family in cancer treatment, several studies have investigated the role of lncRNA in ANTs mechanisms. There are studies on genome-wide lncRNA profiles in DOX-resistant breast cancer cells [15], as well as on lncRNAs related to the pathological response in the DOX neoadjuvant chemotherapy in breast cancer [16]. A specific lncRNA, *H19*, has even been highlighted as a major mediator in breast cancer chemoresistance after DOX treatment [17]. While several studies have explored the lncRNA functions in the ANT mechanisms of action, few studies have initially focused on the lncRNA functions in ANT-induced cardiotoxicity. A study in mice indicated that the up-regulation of lncRNA *FOXC2-AS1* can protect cardiomyocytes from DOX toxicity [18]. Another study showed that the down-regulation of the lncRNA cardiac hypertrophy-related factor (*CHRF*) can diminish cardiac dysfunction and injury [19]. These studies have advised on the critical role of lncRNAs in ANT-induced cardiotoxicity. However, they mostly focus on some targeted lncRNAs and are limited to DOX treatment. The majority of lncRNAs still await further investigation.

In this study, we explored the transcriptomic-wide lncRNA profiles under different ANT treatments. Human cardiac microtissues were exposed to 3 ANT analogs, namely DOX, EPI, and IDA, with therapeutic and toxic doses in triplicated samples across 2 weeks. This study revealed more insights into the alterations of lncRNAs not only during DOX treatment but also during EPI and IDA treatment. By using the transcriptomic-wide approach, this study provided a broad view of lncRNA candidates that are influenced by ANT treatments. We also compared the expression of the lncRNA candidates in the *in vitro* samples to their expression in the cardiac biopsy samples from heart failure patients. These lncRNAs can lead to further research on their potential roles in adverse ANT side effects related to heart disease progression.

## 2 Material and methods

This study used data from the Hepatic and Cardiac Toxicity Systems modelling project funded by the European Union Seventh Framework Programme (FP7/2007–2013).

### 2.1 *In vitro* samples

This study used a human 3D cardiac microtissue model which comprised of 4000 iPSC-derived human cardiomyocytes from a female Caucasian donor and 1000 cardiac fibroblasts from a male Caucasian donor (InSphero). The human cardiac microtissues were cultured in 3D Insight™ Human Cardiac Microtisues Maintenance Medium (InSphero) [20].

The human microtissues were exposed to either a therapeutic or toxic dose of DOX, EPI, and IDA. For each ANT, the therapeutic dose was based on the common clinical treatment dose, while the toxic dose was the IC20 value as determined after one week of exposure [20]. Physiologically-based pharmacokinetic models were applied to calculate the changing of drug concentrations in the interstitial cardiac tissue in 24 h after an oral administration of the particular ANT dose [21]. Thereafter, the experimental design adjusted these ANT dynamic concentration profiles to 3 time periods comprising 0–2 h, 2–8 h, and 8–24 h intervals (Table S1). Within 2 weeks, the medium was renewed 3 times on working days with drug concentration corresponding to these three time periods [22]. ANTs were dissolved in DMSO 0.1% as stock solutions before diluting to the particular drug concentrations. Therefore, the DMSO concentration in the medium fluctuated, and the control samples were exposed to this fluctuating DMSO profile (Table S1). During these 2 weeks of exposure, the microtissues were collected in triplicate at 2, 8, 24, 72, 168, and 240 h of exposure. The microtissues exposed to ANT therapeutic doses were also collected in triplicated after 336 h of exposure.

### 2.2 Biopsy samples

The cardiac biopsies were collected from heart failure patients (n = 31). The investigation conform to the principles outlined in the Declaration of Helsinki. The patient biopsies collection was approved by the Medical Ethics Committee of Maastricht University Medical Center. Informed consent has been obtained from all the subject [20]. The participants consisted of heart failure patients who have no cancer history, cancer survivors who underwent chemotherapy with ANTs, and cancer survivors who underwent chemotherapy without ANTs (Table S2). The biopsies were divided into 3 batches to run the RNA sequencing [20].

### 2.3 RNA sequencing

Total RNA in each sample was isolated using Qiagen AllPrep DNA/RNA/miRNA Universal Kit (Cat #80224). Ribosomal RNAs were depleted by using the Illumina RiboZero Gold kit (Cat #MRZG12324), then samples were prepared by the Lexogen SENSE total RNA library preparation kit (Cat #009.96). The RNA quality and quantity of the samples were checked by the Agilent 420 TapeStation and the Qubit™ before they were sequenced by an HiSeq2000 with 100bp paired-end read [22].

The adapter sequences of the paired-end sequenced raw data were removed by using Trimmomatic version 0.36 [23]. The sequencing quality of samples was examined by FastQC version 0.11.7 [24], and summarized by MultiQC [25] before and after trimming the reads. Two samples (DOX_Tox_240_2 and IDA_The_240_3) had less than 5 million read counts and were excluded from further analysis. The reads were mapped onto the Ensembl human genome reference, version GRCh38.p12, Ensembl Archive Release 93 [26] using RSEM version 1.3.1 [27], and Bowtie2 version 2.3.4.1 [28] with the paired-end option.

### 2.4 Differentially expressed genes in the *in vitro* RNA sequencing data

The raw read counts of all remaining *in vitro* samples were normalized using the DESeq2 R package [29]. The ImpulseDE2 package [30] performed a time-series differential expressed gene analysis between the ANT-treated and the control samples for the 2 weeks period. ImpulseDE2 has its internal DEseq2 normalization, thus, the raw gene read counts from RSEM were used as input data. The function “runImpulseDE2” was applied to perform case-control analysis using gene read count from ANT-treated samples and control samples (p-adj < 0.01). We detected genes which were differentially expressed across all the ANT-treated conditions compared to control samples. The pathway analysis for these differential expressed genes was performed by using ConsensusPathDB [31] with all detected Ensembl gene IDs as the background gene list. A list of lncRNAs, which are related to heart diseases, was extracted from LncRNADisease_v2.0 [32] using the queries “heart” and “cardi” (searching for “cardio”, and “cardiac”). The functions of the lncRNAs related to heart disease were also extracted from the LncTarD database, a manually-curated database of experimentally-supported functional lncRNA–target regulations in human diseases [33], with Ensembl IDs of differential expression lncRNAs as input.

### 2.5 Differentially expressed genes in the biopsies RNA sequencing data

The patient characteristics recruited in batch 3 differed from those in batch 1 and 2; thus the biopsies in batch 3 were excluded (Figure S1). The biopsy samples of batch 1 and 2 (n = 19) were used for further analysis, including heart failure control patients (n = 8), cancer survivors who were treated with ANTs (n = 9), and cancer survivors who were treated without ANTs (n = 2) (Table S2) [20]. The read counts of the biopsy samples were normalized and analyzed by using the DESeq2 package [29] to identify differentially expressed genes between ANT-treated patients (n = 9) and control subjects (n = 8) (p-adj <0.01).

Based on the metadata, there were 7 matched pairs between heart failure control patients and cancer survivors, who were treated with ANTs and developed heart failure as a treatment side-effect [20]. The differences in gene expression of these pairs’ subjects were used to calculate the log2 fold change values in biopsy samples.

### 2.6 Analyses tools

The data analysis was performed in R version 4.0.2 (released on 2020-06-22) [34], using the aforementioned R packages and several R visualization packages including the Upset [35], Tidyverse [36], and ggplot package [37]. The human genome database from the Ensembl Biomart website (https://m.ensembl.org/biomart) [26] was used to annotate the gene type, gene name, and gene function.

## 3 Results

### 3.1 A general view

**Fig. 1:**
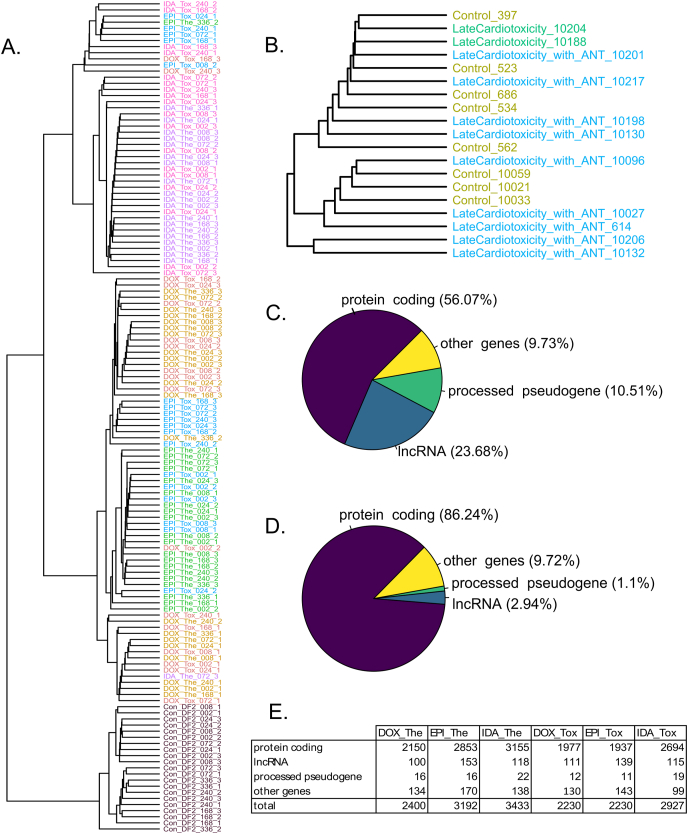
A general view of transcriptome profiles of the *in vitro* and biopsy samples. (A) The cluster tree of the *in vitro* samples' transcriptome profiles. (B) The cluster tree of the biopsy samples' transcriptome profiles. The biopsy samples included heart failure patients without a cancer history (Control) and heart failure patients who underwent cancer treatment with ANT (LateCardiotoxicity_with_ANT) and without ANT (LateCardiotoxicity). The ending numbers are patient IDs (C) The gene type of expressed genes (the average read count across samples >0, annotated using the Ensembl database) in the *in vitro* samples. (D) Gene types of overlapped differentially expressed genes (545 genes) across all *in vitro* ANT-treated conditions compared to control samples. (E) The number of differentially expressed genes in each *in vitro* ANT-treated condition compared to control samples. P-adj <0.01. Con_DF2: control samples; DOX: doxorubicin, EPI: epirubicin, IDA: idarubicin; The: therapeutic dose, Tox: toxic dose; 002, 008, 024, 072, 168, 240, 336 are corresponding exposure periods; ANT: anthracycline(s).

Human 3D cardiac microtissues were exposed to either therapeutic dose or toxic dose of DOX, EPI, and IDA for two weeks, and harvested in triplicated after 2 h–336 h of exposure (Table S1). After removing 2 samples with a low read count (<5 million), 145 *in vitro* samples were used for transcriptome-wide analysis. A cluster tree of the *in vitro* transcriptome profiles demonstrated a clear separation between control samples and ANT-treated samples, in which all control samples were grouped in one branch of the cluster tree (Fig. 1A). In the ANT-treated samples sub-branch of the cluster tree, most of the DOX and EPI-treated samples were specifically grouped in one sub-branch, whereas the IDA-treated samples were mainly grouped in another sub-branch (Fig. 1A). By contrast, the transcriptome profiles of the biopsies data (n = 19) did not demonstrate a clear distinction between the heart failure control patients and the heart failure patients who underwent ANT treatments (Fig. 1B).

While the human genome contains 58,395 detectable genes, 31,910 genes were expressed in the *in vitro* cardiac tissues under ANT exposure conditions with the average read count across samples is larger than 0. According to the Ensembl database, these active genes belong to different gene types (Fig. 1C). Protein-coding genes, as key components of the cellular mechanisms, contributed to a large proportion of genes that were expressed (56.07%). Notably, lncRNA genes, which lack the protein-coding ability, accounted for 23.68% of genes that were expressed in both ANT-treated and control samples. The rRNA genes, which is a predominant RNA group, were rarely detected as ribo-depleted total RNA library prepration was used in this study.

### 3.2 Differentially expressed (DE) genes

By using the ImpulseDE2 tool on the *in vitro* data, DE genes (adjusted p-value<0.01) were detected in each ANT-treated condition compared to the control condition during the 2 weeks of exposure. In total, 545 genes were consistently differentially expressed in all *in vitro* ANT-treated conditions compared to control. Most of these overlapping DE genes were protein-coding genes, while 16 genes (2.94%) were lncRNA genes (Fig. 1D). In particular, each ANT-treated condition had over 100 lncRNA genes which were differentially expressed compared to the control samples (Fig. 1E).

**Fig. 2:**
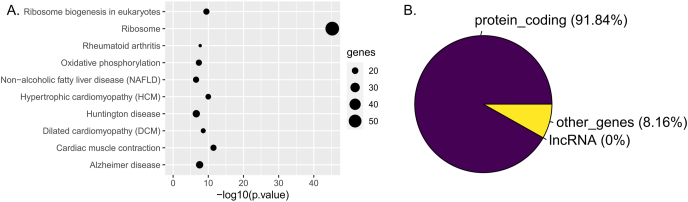
Pathway analysis outcomes from overlapped differentially expressed (DE) genes in the *in vitro* anthracycline-treated samples. (A) Top 10 over representative pathways of the 545 overlapped DE genes across *in vitro* ANT-treated conditions; (B) Gene type proportions of the overlapped DE genes that popped up in the pathway analysis.

Pathway analysis revealed which cardiac functions the 545 overlapped DE genes are involved in. Three of the top 10 over representative pathways are heart disease pathways; it emphasizes that the effect of ANT treatment through these genes might facilitate cardiotoxicity and heart failure development (Fig. 2A, Table S3). A previous study using the same dataset with another differential gene expression analysis tool and other pathway databases also provided a similar outcome [20]. Although pathway analysis can capture a part of the ANT toxicity mechanism, its expository ability is mainly restricted to protein-coding genes (Fig. 2B). The 3 heart disease pathways of the top 10 over representative pathways are from the KEGG database and contain only protein-coding genes (Table S3) [38]. This conventional approach neglects non protein-coding genes, especially lncRNAs which were consistently differentially expressed between ANT-treated conditions and control (Fig. 1D).

In the biopsies data, 37 DE genes, including 5 lncRNA genes, were detected by DESeq2 between ANT-treated patients and control subjects. The pathway analysis did not reveal any connection of these 37 DE genes to heart function and heart disease (Table S4). Of the 5 DE lncRNA genes in biopsies data, the *LINC00612* gene might be involved in acute myocardial infarction [39], while the AL031280.1 gene was also differentially expressed in IDA-treated samples compared to control.

### 3.3 Differentially expressed lncRNAs

Sixteen lncRNA genes were consistently differentially expressed in all ANT-treated conditions while other lncRNAs were only differentially expressed at certain ANT-treated conditions (Table 1). For example, 6 lncRNAs were differentially expressed only in the DOX-treated conditions, while 13 lncRNAs were specifically differentially expressed in the EPI-treated conditions. The IDA-treated conditions had the highest number of drug-specific lncRNAs with 17 DE lncRNAs (Table 1). For the dose-specific effects, the *AL451123.1* lncRNA was differentially expressed in all ANT samples treated with the therapeutic dose. The other 2 lncRNAs, *AL133453.1* and *BACE1-AS*, were differentially expressed in all ANT samples treated with the toxic dose.

**Table 1:** Differentially expressed (DE) lncRNAs in different anthracycline (ANT) treatment conditions.

	DOX-treated condition	EPI-treated condition	IDA-treated condition
Numbers of DE lncRNA	6	13	17
LncRNAs	*AC025259.1*, *AL031985.3*, *FP671120.5*, *AD000090.1*, *HCP5*, *FTX*	*AC055811.1*, *N4BP2L2-IT2*, *AC078880.4*, *AC009264.1*, *AL132656.2*, *AC007262.2*, *LINC02503*, *AL354733.3*, *XIST*, *AC092828.1*, *AP000766.1*, *AC125257.1*, *AC007114.2*	*AC009779.2*, *AL162311.3*, *AC009133.2*, *ESRG*, *AC100803.3*, *AC104794.2*, *OVCH1-AS1*, *C4B-AS1*, *SNHG22*, *LINC02108*, *URB1-AS1*, *SNHG16*, *C4A-AS1*, *KCNQ1OT1*, *AC093495.1*, *AC018761.2*, *GABPB1-AS1*
Numbers of DE lncRNA	16		
LncRNAs	*AC006064.4*, *AC007009.1*, *RMRP*, *LINC00622*, *SNHG7*, *AC093866.1*, *SNHG29*, *H19*, *AC132217.1*, *AC124312.3*, *LINC01638*, *AC020909.3*, *AC106791.1*, *BDNF-AS*, *AC010680.5*, *PCAT19*		

Note: DOX: doxorubicin, EPI: epirubicin, IDA: idarubicin. Each treatment condition consists of samples treated with corresponding drugs in therapeutic dose and toxic dose.

**Fig. 3:**
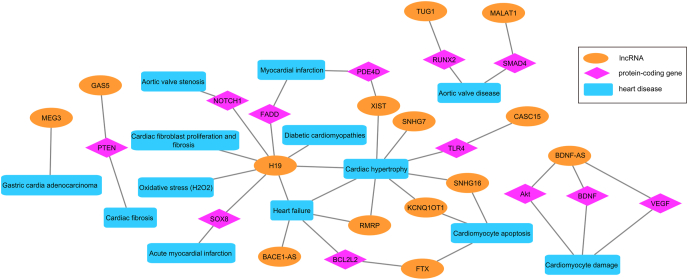
Network representing relationships between differentially expressed (DE) lncRNAs and corresponding heart diseases.

The LncTarD and LncRNADisease_v2.0 databases were used to explore the association between the DE lncRNAs and heart disease. The LncTarD database collects functional lncRNA–target regulations in humans [33], while the LncRNADisease_v2.0 is a lncRNAs related diseases database [32]. Through these databases, the association of DE lncRNAs with heart disease was detected, especially the *H19* and *FTX* genes were mentioned in both databases (Table S5, S6). Furthermore, other studies also highlighted the potential causal relationships between the DE lncRNAs and heart diseases [[40], [41], [42], [43], [44]]. Based on this prior knowledge, a network was established to represent the relationship between DE lncRNAs and corresponding heart diseases (Fig. 3). This network highlights some key lncRNAs, such as *H19* and *BDNF-AS*, which strongly connect to cardiac diseases.

**Fig. 4:**
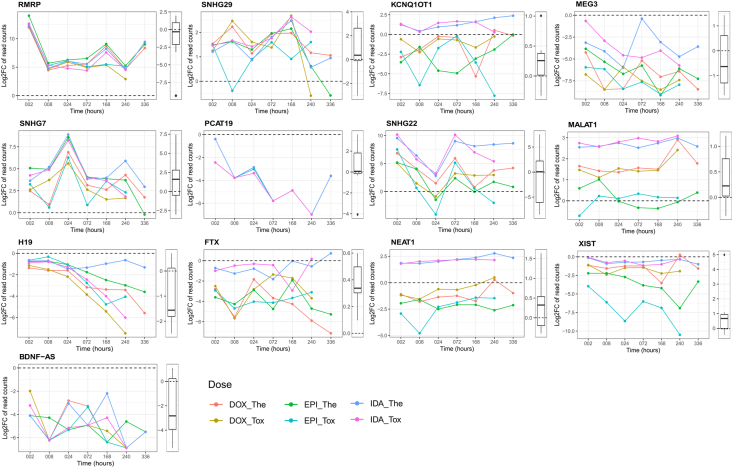
Log2 fold change (log2FC) gene expression in selected differentially expressed (DE) lncRNAs. For each lncRNA, log2FC values over time between ANT-treated and control samples in the *in vitro* data are presented in the line chart, while the distribution of the log2FC values between ANT-treated patients and control patients in matched pairs are represented in the boxplot. Con_DF2: control samples; DOX: doxorubicin, EPI: epirubicin, IDA: idarubicin; The: therapeutic dose, Tox: toxic dose; 002, 008, 024, 072, 168, 240, 336 are corresponding exposure periods in hours; ANT: anthracycline.

Of the 16 DE lncRNAs found in all ANT-treated conditions, some lncRNAs showed a remarkable alteration in their expressions (Fig. 4). *SNHG29*, *SNHG7*, and *RMRP* were up-regulated in the *in vitro* ANT-treated conditions. Although these lncRNAs’ expressions were not statistically different between patient groups in the biopsies data, the expression of *SNHG29* and *SNHG7* was also up-regulated in the ANT-treated patients compared to the control subjects (Fig. 4). Other lncRNAs that are *H19*, *BDNF-AS*, and *PCAT19* were down-regulated in the *in vitro* ANT-treated conditions, especially, the expression of *H19* showed a clear distinction between ANT-treated conditions after a longer exposure time. Similarly, the expressions of *H19* and *BDNF-AS* genes were down-regulated in the ANT-treated patients (Fig. 4).

Other lncRNAs were differentially expressed in specific *in vitro* ANT-treated conditions (Table 1). For instance, the lncRNA *XIST* was prominently down-regulated in the EPI-treated conditions, whereas the *FTX* gene was down-regulated in the DOX-treated conditions. The 2 lncRNAs, *SNHG22* and *KCNQ1OT1*, were only significantly up-regulated in the IDA-treated conditions (Fig. 4). Additionally, *MEG3* was significantly down-regulated in the DOX and EPI-treated conditions, while *MALAT1* was significantly up-regulated in the DOX and IDA-treated conditions. *NEAT1* is an interesting lncRNA, which showed increases expression in the IDA-treated condition and decreased expression in the EPI-treated condition, while its expression in the DOX-treated condition was not differentially expressed compared to the control samples (Fig. 4). The expressions of these lncRNAs also differed between ANT-treated patients and control subjects in the biopsies data (Fig. 4).

## 4 Discussion

While ANT is an important drug family for cancer chemotherapy, its adverse effects can damage cardiomyocytes, cause cardiac dysfunction, and lead to heart failure. While DOX was discovered in the 1960s and is considered as the first generation of ANTs; EPI and IDA were developed from DOX and daunorubicin respectively, and are considered as the second generation of ANTs [3]. Early studies have emphasized that EPI and IDA might be less cardiotoxic than the former ANT generation. However, a recent cohort study with nearly 30,000 cancer survivors manifested that the cardiotoxicity of both EPI and IDA could be equivalent to DOX [45]. Consequently, it is necessary to elaborate on the ANT-induced cardiotoxicity, not only the DOX toxic mechanism but also the toxic mechanism of other ANT analogs. This study demonstrated that the *in vitro* transcriptome-wide analysis can capture the cardiotoxicity which was caused by DOX, EPI, and IDA. Particularly, this study revealed the differential expression of lncRNAs, which can be potential targets for ANT-induced cardiotoxicity.

The *in vitro* transcriptome profiles of the ANT-treated samples differed from those of control samples, especially the transcriptome profiles of ANT-treated samples were mainly grouped based on their drug treatment (Fig. 1A). The gene expression profiles of the DOX-treated samples were similar to those of the EPI-treated samples, rather than those of the IDA-treated samples. This phenomenon is supported by the prior knowledge that 10.13039/100013832EPI, as a derivative from DOX, can share a similar mechanism of action with DOX [3]. By contrast, IDA is an analog derived from daunorubicin and is more lipophilic than DOX [46]. This underlying chemical difference could possibly explain why IDA treatments cause different gene expression profiles in cardiac tissue compared to DOX and EPI treatments. Hence, the *in vitro* transcriptome analysis could indicate the subtle distinction in mechanism between specific ANTs. However, some of the therapeutic-treated samples were grouped with the toxic-treated samples, specifically for IDA and DOX treatment (Fig. 1A). We analyzed the proteomic profiles of these *in vitro* ANT-treated samples [47], and have seen a clear separation between the therapeutic and toxic dose at the proteomic level. Thus, it seems that there are more disruptions in the transcriptome than in the proteome.

In the *in vitro* experiment, although a part of DE genes were protein-coding genes, another part of the DE genes were lncRNA genes (Fig. 1D–E, Table 1). Specifically, 16 lncRNA were consistently differentially expressed across all ANT-treated conditions compared to control samples in a two-week exposure (Table 1). While the conventional pathway analysis can demonstrate how DE protein-coding genes relate to heart disease (Fig. 2A), it neglects the role of DE lncRNAs in the ANT-induced cardiotoxicity, in which no lncRNA gene was recognized on the pathway databases (Fig. 2B). Therefore, clarification of DE lncRNA functions is needed for a better understanding of the ANT toxicity mechanism, especially when lncRNA genes have recently emerged as potential targets in ANT-related studies [[15], [16], [17], [18]].

The overlapping DE 16 lncRNAs across *in vitro* ANT-treated samples suggests that the expression of these lncRNAs could be affected by typical ANTs mechanisms, although these lncRNAs did not appear in the known heart disease or other cellular pathways using the conventional pathway analysis (Fig. 2). Nevertheless, using lncRNA databases and doing literature research provided the function of not only the 16 overlapping DE lncRNAs but also other lncRNAs, which were differentially expressed in particular ANT-treated conditions (Fig. 3, Table S5, S6).

Both lncRNA databases manifested the role of *H19*, one of the 16 overlapping DE lncRNAs, in several heart diseases (Fig. 3, Table S5, S6). *H19* was down-regulated in all *in vitro* ANT-treated conditions as well as in the ANT-treated patients in the biopsy samples (Fig. 4), possibly due to oxidative stress. This phenomenon corresponds with another observation that showed a reduction in the *H19* level as a response to oxidative stress (H_2_O_2_) in C-kit + cardiac progenitor cells [40]. This study also indicated some conserved binding sites of *miR-675*, a miRNA generated from *H19*, on 3′UTR of *USP10*; and the *H1*9/miR*‐675/USP10* axis can suppress both p53 and p21 expression [40]. Furthermore, *H19* can suppress the activity of *miR-22-3p* by binding to a *miR-22-3p* binding site in the *KDM3A* gene. An *H19* upregulation can then improve cardiac performance, alleviate cardiac fibrosis, and decreasing inflammation [41]. These studies have encouraged that *H1*9 might be a potential biomarker and therapeutic target for ANT-induced cardiotoxicity. However, the overexpression of *H19* can contribute to the chemoresistance of breast cancer cells [17]. This suggests that *H19* can be involved in the on-target toxicity of ANTs, in which down-regulated *H19* produces desired treatment response in tumors, but lead to cardiotoxicity in cardiac cells.

Other overlapping DE lncRNAs are also involved in heart diseases and cellular mechanisms. Both *H19* and *RMRP* engage in cardiac hypertrophy and heart failure progression [42], while a *BDNF-AS* downregulation can activate *BDNF*, *VEGF*, and *Akt*, and thus rescue hypoxia/reoxygenation-induced damage in cardiomyocyte [43] (Fig. 3). Hence, the *BDNF-AS* was downregulated both in the *in vitro* experiment and in the ANT-treated patients (Fig. 4), which suggests the recovery intention of cardiac tissue under ANT treatment. Another lncRNA, namely *SNHG7*, has also emerged as a novel regulator for cardiac hypertrophy (Fig. 3). *SNHG7* was up-regulated in the *in vitro* ANT-treated samples as well as ANT-treated patients (Fig. 4), while a study in neonatal rat cardiomyocytes revealed that the up-regulation of the *SNHG7* genes can stabilize *SDAD1* mRNA, and then facilitate cardiac hypertrophy [44]. Other lncRNAs, including *SNHG29* and *PCAT19*, have not been investigated in heart failure contexts, but they are known as potential regulators in cell signaling pathways. *SNHG29* acts as a competing endogenous RNA, in which it sponges the *miR-223-3p* to regulate the *CTNND1* expression. Therefore, *SNHG29* can modulate the Wnt/β-catenin signaling pathway via the *miR-223-3p/CTNND1* axis [48]. *PCAT19* also acts as a competing endogenous RNA, in which it sponges the *miR-182* to regulate the *PDK4*, and consequently modulates the glycolysis and mitochondrial respiration in laryngeal cancer cell lines [49]. Additionally, the *PCAT19* expression was negatively correlated with the p53 expression in non-small cell lung cancer patients, and the silencing of *PCAT19* elevated the p53 expression level in H1993 cells [50]. These aforementioned studies have demonstrated the abilities of the overlapping DE lncRNAs related to cellular functions and heart disease, as well as their potential for ANT-induced cardiotoxicity research.

Some lncRNAs were only differentially expressed in samples treated with particular ANT doses. For example, the *AL451123.1* was differentially expressed in all ANT therapeutic-treated samples, whereas the *AL133453.1* and *BACE1-AS* were differentially expressed in all ANT toxic-treated samples. The functions of *AL451123.1* and *AL133453.1* lncRNAs are still unclear, whereas *BACE1-AS* is known as a heart failure-related lncRNA [32] (Fig. 3). The *BACE1-AS* up-regulation might increase the *BACE1* level and accumulate β-amyloid, which is *BACE1*'s product. This dysregulation of the *BACE1-AS/BACE1*/β-amyloid axis could diminish the cardiomyocyte viability [51].

Similarly, other lncRNAs were differentially expressed in samples treated with particular ANT analogs. Of the 6 lncRNAs which were differentially expressed in DOX-treated samples, the lncRNA *FTX* could inhibit apoptosis and reduce hypertrophy in cardiomyocytes [52,53], while *AD000090.1* was proposed to regulate hypoxic responses [54] (Fig. 3). LncRNA *XIST* was only differentially expressed in EPI-treated samples and could promote the progression of cardiac hypertrophy resulting in heart failure disease [55] (Fig. 3). *KCNQ1OT1* and *SNHG16* were notable lncRNAs, which were differentially expressed in IDA-treated samples. Several studies have advocated that these 2 lncRNAs may facilitate cardiomyocyte apoptosis and accelerate cardiac hypertrophy [56,57]. The lncRNA databases also recommend other heart disease-related lncRNAs, which were differentially expressed in particular ANT-treated conditions, including *MALAT1, MEG3, TUG1, GAS5, CASC1S* (Fig. 3, Table S5, S7). When heart disease-related lncRNAs were atypically affected by certain ANT-treated conditions, they could reveal the subtle difference in toxic mechanisms of specific ANT analogs.

Although the DE lncRNAs in specific *in vitro* ANT-treated conditions could release the mechanism of individual ANT analogs, it is an obstacle to confirm this knowledge in clinical application. In this study, the transcriptome profiles of the biopsies data (n = 19) did not demonstrate a clear distinction between the heart failure control patients and the heart failure patient who underwent cancer treatments (Fig. 1B). This result is consistent with the previous proteomic study [47], in which there was no apparent difference in proteomic profiles between ANT-treated patients and control subjects. There were 37 DE genes between the ANT-treated patients and control groups; however, there was no clear relation between these DE genes and cardiac function. One reason for this could be that all participants were heart failure patients and non-significant differences related to heart disease were found among them. Furthermore, cancer patients often underwent chemotherapy with multi-drug combinations to improve the treatment efficiency [58,59]. Most patients involved in this study had been treated with multiple ANT analogs and other anti-tumor drugs a long time ago (Table S2). Although it is difficult to distinguish the effect of ANT analogs leading to cardiovascular disease in a clinical setting, we observed some similar changes in lncRNA expressions between the *in vivo* experiments and human biopsies (Fig. 3).

## 5 Conclusion

This study provided new insight into the transcriptome alterations related to ANT-induced cardiotoxicity, especially the differential expression of lncRNAs. While the conventional pathway analysis might not be able to capture the role of these DE lncRNAs in cellular mechanisms, recent research has acknowledged the involvement of these lncRNAs in heart disease progression (Fig. 3). *H19* seems to be involved in both chemoresistances as well as cardiotoxicity, which suggests its participation in the on-target toxicity of ANTs. Some lncRNAs, including *H19*, *RMRP*, *BDNF-AS*, and *SNHG7*, could be targets for further research on the typical mechanisms of ANT-induced cardiotoxicity, while other lncRNAs could advocate the cardiac responses to certain ANT analogs and doses. Although the functions of some lncRNAs have been explored, further study is needed to investigate the functionalities of other unknown lncRNAs (Table 1) related to heart disease.

## Funding

This work was supported by the 7th Framework Programme of the 10.13039/501100000780European Union (FP7/2007–2013) [No. 602156] for the HeCaToS project.

## Declaration of competing interest

The authors declare that no conflicts of interest exist.

## Data accessibility

The *in vitro* raw RNA sequencing data are available in the BioStudies database (http://www.ebi.ac.uk/biostudies). The accession number of the RNAseq data from DOX, EPI, and IDA *in vitro* samples are S-HECA10, S-HECA11, and S-HECA12, respectively. The accession number of the RNAseq data from biopsy samples is S-HECA469.

The code of the data analysis is available in Github (https://github.com/NhanNguyen000/lncRNA_ANT).

## CRediT authorship contribution statement

**Nhan Nguyen:** Conceptualization, Methodology, Investigation, Formal analysis, Writing – original draft. **Terezinha Souza:** Conceptualization, Writing – review & editing. **Jos Kleinjans:** Writing – review & editing, Supervision, Funding acquisition. **Danyel Jennen:** Writing – review & editing, Supervision.

## Appendix A Supplementary data

figs1 figs1
