# Supplementary figures and images for: Transcriptome analysis of long noncoding RNAs reveals their potential roles in anthracycline-induced cardiotoxicity
Source: Noncoding RNA Res. 2022 Jan 23;7(2):106–13. doi: 10.1016/j.ncrna.2022.01.002 (PMC8967700; doi:10.1016/j.ncrna.2022.01.002)

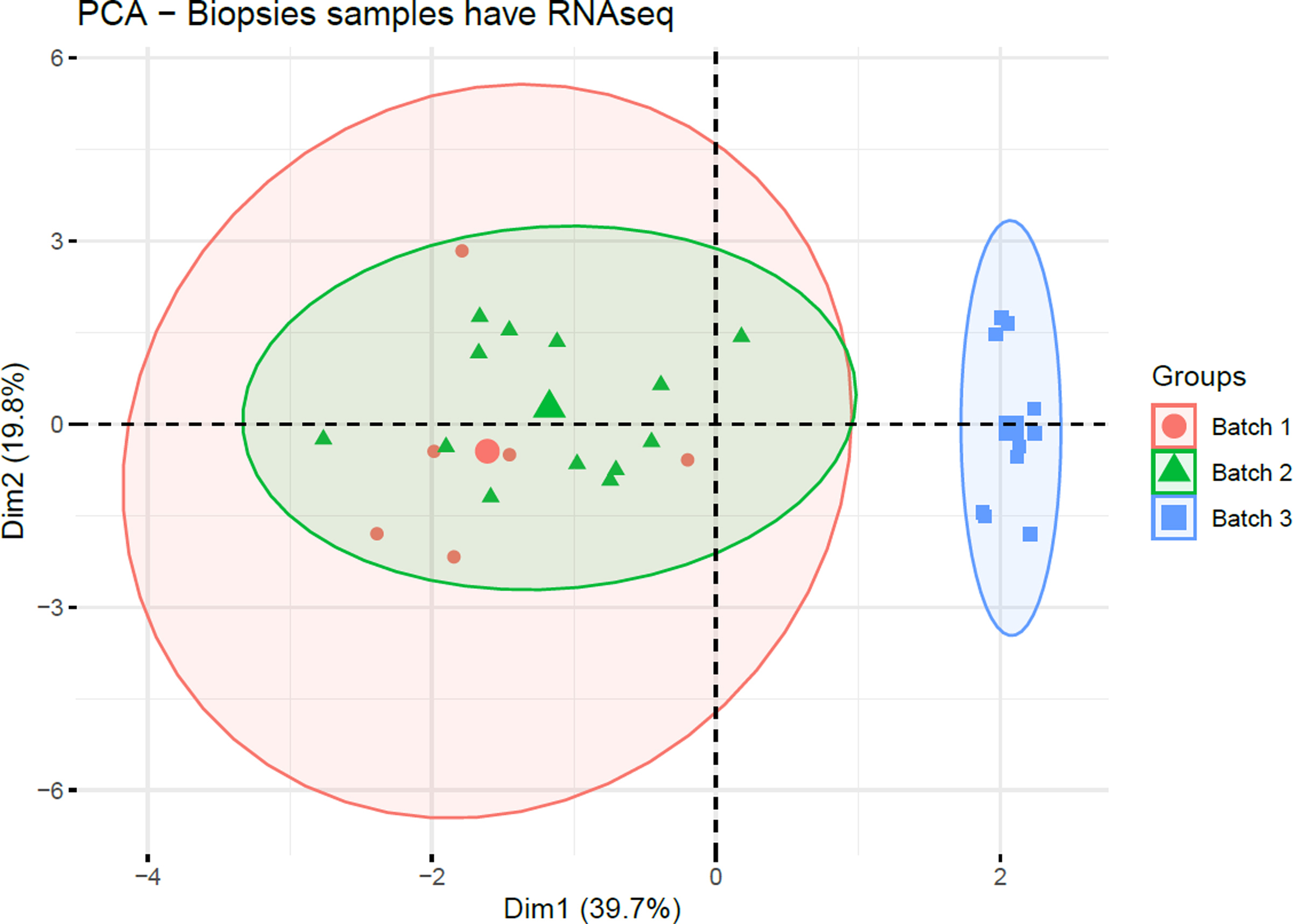

Supplement: figs1 [file mmcfigs1.jpg]
